# Vermont Medical Society—Proceedings at the Annual Meeting, Oct. 16, 1844

**Published:** 1845-01

**Authors:** Z. P. Burnham

**Affiliations:** Secretary


*Vermont Medical Society.*—The Vermont State Medical Society met at the State House, in Montpelier, October 16, 1844, agreeably to the By-laws. Several addresses upon medical subjects were delivered before the Society.

The following persons were elected officers for the year ensuing:

Anderson G. Dana, Brandon, President; Horace Eaton, Enosburgh, Vice President; Z. P. Burnham, Montpelier, Secretary; J. A. Allen, Middlebury, Corresponding Secretary; James Spalding, Montpelier, Treasurer.

Charles Hall, Orange Smith, Eldad Alexander, John Dewey, B. R. Palmer, W. R. Ranney, James Tinker, Noadiah Swift, J. B. Porter, J. Rice, H. H. Reynolds, John L. Chandler, Walter Carpenter, Censors.

Drs. J. A. Allen and A. G. Dana, were appointed delegates to attend the examination of Students at Castleton Medical College.

Drs. W. R. Ranney and J. Y. Dewey were appointed delegates to the Woodstock Medical Institution. Z. P. Burnham, *Secretary.*

*Ibid.*

